# A case of a giant lipidized dermatofibroma

**DOI:** 10.1016/j.jdcr.2025.02.041

**Published:** 2025-03-22

**Authors:** Craig M. Fisher, Carolyn A. Robinson, Kevin J. Krauland

**Affiliations:** a Department of Dermatology, Wilford Hall Ambulatory Surgical Center, JBSA Lackland, Texas; b Department of Pathology, Brooke Army Medical Center, JBSA Fort Sam Houston, Texas

**Keywords:** dermatofibroma, fibrohistiocytic, giant dermatofibroma, lipidized dermatofibroma

## Introduction

Dermatofibromas, also known as benign fibrous histiocytomas, are benign fibrohistiocytic proliferations of unclear etiology usually originating in the dermis.^1^ They commonly present as slow-growing nodules on the extremities with a predilection for the lower extremities but may present on any skin surface. Rarely do dermatofibromas exceed 2 cm in diameter. Dermatofibromas can follow a variety of histopathologic patterns including cellular, hemosiderotic, aneurysmal, lipidized, epithelioid, clear cell, atrophic, common type, and other less commonly described subtypes.^2^ Common histologic features consist of a predominantly dermal-based proliferation of spindled-to-epithelioid cells with variable amounts of cytologic atypia and pleomorphism with collagen trapping at the periphery and overlying epidermal induction. Effacement of the rete ridges is common and may exist with or without a Grenz zone. The tumor may extend into the subcutaneous fat and can demonstrate focal areas of fat necrosis, which can help to distinguish it from dermatofibrosarcoma protuberans.^3^ Touton giant cells and ringed lipidized siderophages may also be present.

Among the rarer clinical variants of dermatofibromas is the giant subtype that must be > 5 cm in clinical size^4^^,^^5^;<30 cases have been reported. The lipidized variant of dermatofibromas demonstrates foamy histiocytes scattered within sclerotic dermal collagen. Lipidized dermatofibromas were initially described as occurring more frequently on or around the ankles giving rise to the informal name “ankle-type” dermatofibroma.^6^ Herein is described a giant lipidized dermatofibroma on the ankle of a 64-year-old man.

## Case report

**Fig 1:**
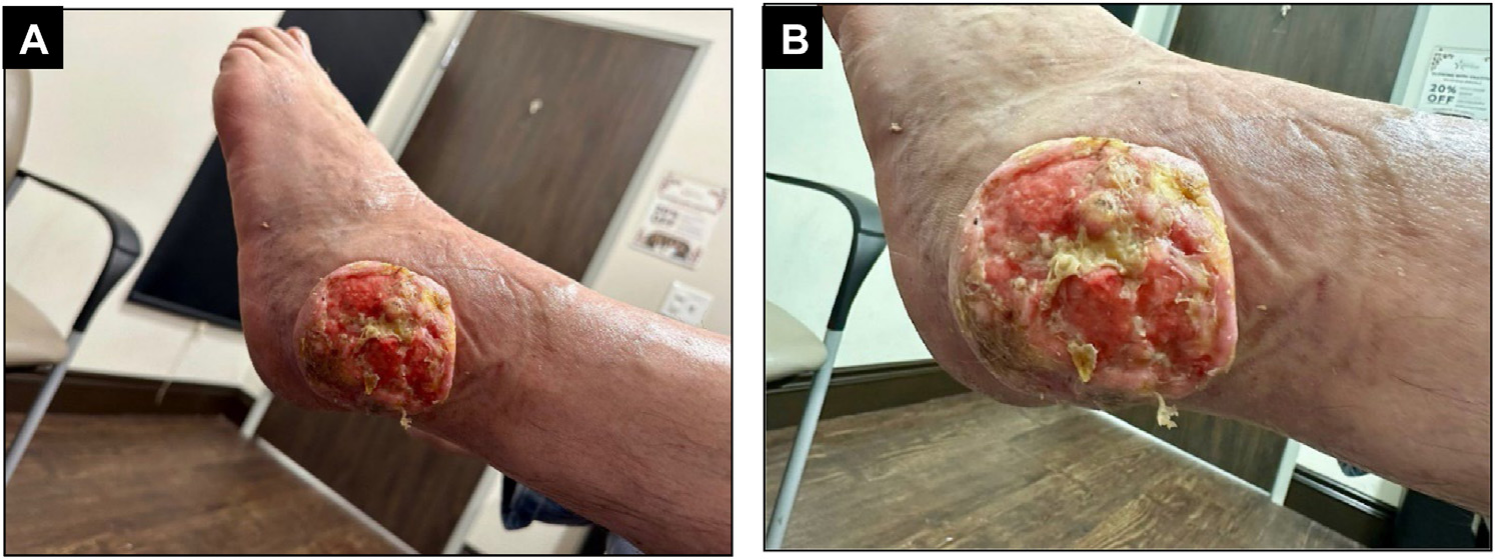
**A,** A 9 cm × 8.5 cm eroded, fungating circumscribed tumor overlying the left lateral malleolus. **B,** The tumor surface was ulcerated and friable. There is a notable peripheral collarette around the tumor.

A 64-year-old Fitzpatrick skin type II man presented with a tumor on his left ankle that had been slowly growing over a period of approximately 12 years. The lesion was not painful; however, it was bothersome because of its size and constant pinpoint bleeding diffusely throughout the lesion's surface. Physical examination revealed a 9 cm × 8.5 cm firm, well-defined, fungating ulcerated tumor without palpable infiltration of deeper structures overlying the left lateral malleolus (Fig 1). The tumor was not entirely sessile but was attached securely only at the central base of the lesion.

A shave biopsy of a representative portion of the lesion demonstrated pseudoepitheliomatous hyperplasia of the epidermis overlying a cellular interstitial proliferation of plump, stellate to spindled fibrohistiocytes within a fibrotic collagenous background with foci of collagen trapping at the periphery of the lesion. Numerous multinucleated cells with admixed vessels were also noted. The patient was referred to a dermatologic surgeon for further management.

**Fig 2:**
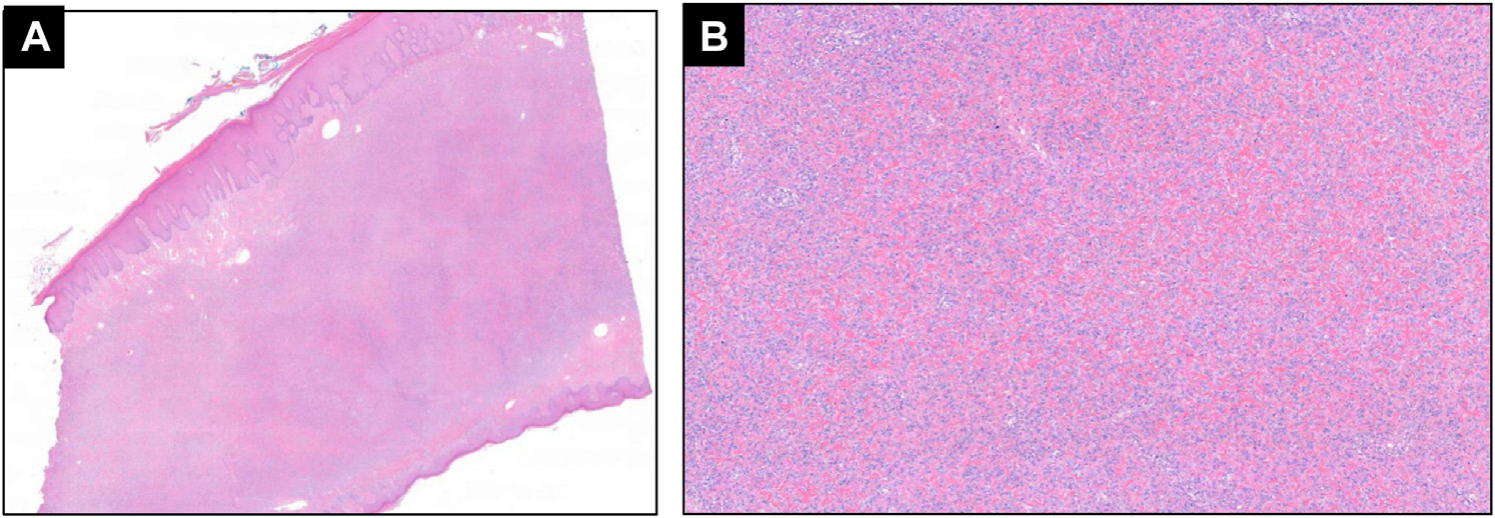
**A,** Excisional biopsy demonstrated a cellular dermal fibrohistiocytic proliferation extending to the reticular dermal-subcutaneous junction with prominent overlying epidermal induction and tabling of the rete ridges with peripheral collagen entrapment. **B,** Cellular dermal proliferation consisting of foamy histiocytes embedded in sclerotic, hyalinized dermal collagen with interspersed foam spindled-to-epithelioid cells. (**A,** Hematoxylin-eosin stain; original magnification: **A,** ×2.)

**Fig 3:**
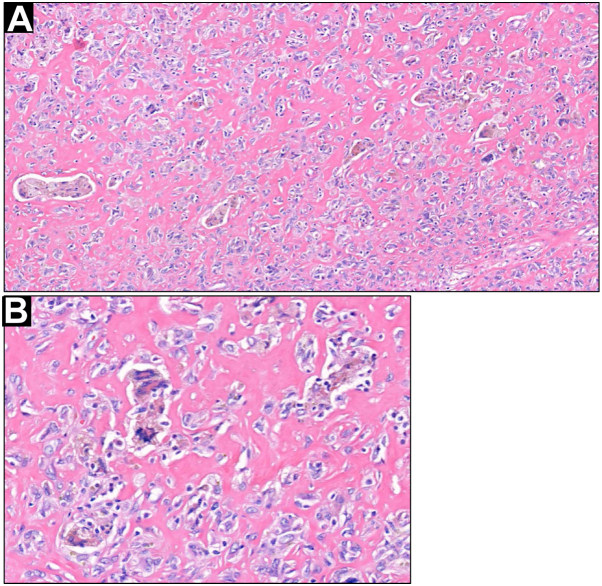
A, Foamy histiocytes, siderophages, and ringed siderophages were noted throughout the biopsy specimen. **B,** Ringed siderophages with intervening hyalinized stroma. (**A** and **B,** Hematoxylin-eosin stain; original magnifications: **A,** ×200; **B,** ×400.)

**Fig 4:**
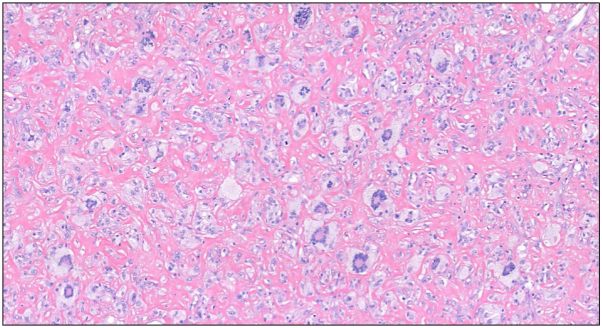
Numerous Touton giant cells within hyalinized stroma and admixed foamy fibrohistiocytic cells.

The patient elected for an excisional biopsy without additional imaging of the lower extremity due to lack of insurance. The excisional biopsy demonstrated similar histologic features throughout the remainder of the lesion including pseudoepitheliomatous hyperplasia (Fig 2) and ulceration, with the underlying dermis containing a poorly circumscribed lesion composed of numerous fibrocytes associated with peripheral collagen trapping, increased vascularity and hemosiderin-laden macrophages (Fig 3) as well as abundant foamy histiocytes (Fig 4).

As the lesion extended to both the peripheral and deep tissue margins the patient was offered re-excision. He declined and opted to monitor clinically for recurrence. The patient was ultimately lost to follow-up.

## Discussion

Dermatofibromas are common benign tumors that favor the lower extremities and follow a variety of histologic patterns. Dermatofibromas are typically <1 cm, uncommonly exceed 2 cm, and rarely surpass 5 cm in size. Dermatofibromas >5 cm in size attain the distinction of giant dermatofibromas, often demonstrate pedunculated growth, and occur most commonly on the lower extremities.^5^ Although clinically concerning for a skin or soft-tissue malignancy, given the large size and possible overlying ulceration, as in our case, the giant subtype displays the same benign clinical behavior of classic dermatofibromas.^7^^,^^8^

Clinically, the differential diagnosis of giant lipidized dermatofibromas includes varieties of cutaneous keratinocyte carcinomas, melanoma, pyogenic granuloma, epithelioid fibrous histiocytoma, and infectious etiologies such as deep fungal, atypical mycobacterial, and protozoal infections. Histologically lipidized dermatofibromas demonstrate variable amounts of foamy histiocytes and sclerotic, hyalinized stroma that can have overlap with entities such as sclerosing epithelioid fibrosarcoma and xanthomas depending on which component is more abundant.^9^ Distinguishing features that favor dermatofibroma are collagen trapping at the periphery, overlying epidermal basaloid or follicular induction, effacement or tabling of the rete ridges, the presence of Touton giant cells, hemorrhage, hemosiderin, admixed inflammatory cells, and ringed lipidized siderophages. None, except for ringed siderophages, are specific to dermatofibromas, but the constellation of features supports the diagnosis.

Additional immunohistochemical staining would reveal positivity for CD163, variable expression of factor XIIIa, and CD68 (although this is a less specific marker of fibrohistiocytic lineage). Ki67 should show elevated proliferative index of dermatofibromas relative to dermatofibrosarcoma protuberans.^10^ Negative stains include CD34, S100, EMA, SMA, and desmin.

Typical dermatofibromas can be observed or treated if symptomatic with excision or destructive measures, including lipidized dermatofibromas. Despite the size of the giant dermatofibromas, there have been no reported recurrences or metastasis after conservative excision.

This case highlights the far end of the spectrum of clinical manifestations of dermatofibromas but reinforces the benign, indolent nature of the tumors despite rare variants reaching giant sizes.

## Conflicts of interest

None disclosed.
